# Spin stress contribution to the lattice dynamics of FePt

**DOI:** 10.1126/sciadv.aba1142

**Published:** 2020-07-08

**Authors:** A. von Reppert, L. Willig, J.-E. Pudell, S. P. Zeuschner, G. Sellge, F. Ganss, O. Hellwig, J. A. Arregi, V. Uhlíř, A. Crut, M. Bargheer

**Affiliations:** 1 Institute of Physics and Astronomy, University of Potsdam, Karl-Liebknecht-Str. 24-25, 14476 Potsdam, Germany.; 2 Helmholtz-Zentrum Berlin für Materialien und Energie GmbH, Wilhelm-Conrad-12 Röntgen Campus, BESSY II, Albert-Einstein-Str. 15, 12489 Berlin, Germany.; 3 Institut für Physik, Technische Universität Chemnitz, Reichenhainer Str. 70, 09126 Chemnitz, Germany.; 4 Institut für Ionenstrahlphysik und Materialforschung, Helmholtz-Zentrum Dresden-Rossendorf, Bautzner Landstrasse 400, 01328 Dresden, Germany.; 5 CEITEC BUT, Brno University of Technology, Purkyňova 123, 612 00 Brno, Czechia.; 6 Institute of Physical Engineering, Brno University of Technology, Technická 2, 616 69 Brno, Czechia.; 7 FemtoNanoOptics Group, Institut Lumière Matière, Université de Lyon, CNRS-Université Lyon 1, 69622 Villeurbanne, France.

## Abstract

Invar-behavior occurring in many magnetic materials has long been of interest to materials science. Here, we show not only invar behavior of a continuous film of FePt but also even negative thermal expansion of FePt nanograins upon equilibrium heating. Yet, both samples exhibit pronounced transient expansion upon laser heating in femtosecond x-ray diffraction experiments. We show that the granular microstructure is essential to support the contractive out-of-plane stresses originating from in-plane expansion via the Poisson effect that add to the uniaxial contractive stress driven by spin disorder. We prove the spin contribution by saturating the magnetic excitations with a first laser pulse and then detecting the purely expansive response to a second pulse. The contractive spin stress is reestablished on the same 100-ps time scale that we observe for the recovery of the ferromagnetic order. Finite-element modeling of the mechanical response of FePt nanosystems confirms the morphology dependence of the dynamics.

## Teaser

Two-pulse laser excitation of FePt nanolayers distinguishes ultrafast stresses of magnetic excitations from the Poisson effect.

## INTRODUCTION

Invar materials exhibit almost zero thermal expansion over a wide temperature range (*1*). Although the discovery of a 10-fold reduction of the thermal expansion of the Fe_0.65_Ni_0.35_ alloy compared to its pure elements (*2*) dates back to 1897, its origin remained an active area of solid-state research over the next century (*3*–*5*). Invar behavior requires a mechanism that counteracts the thermal expansion resulting from anharmonic phonon-phonon interactions. For magnetic invar materials, it is found that the required contractive stress originates from an increased volume for the spin-ordered state compared to the disordered state that can now be predicted in different ab initio approaches (*4*, *5*). Quantitative, time-resolved studies of the structural dynamics have recently started to explore the response of the lattice to magnetic stresses (*6*–*12*), which are attributed to the transfer of angular momentum (*6*, *7*), energy (*8*, *12*), and entropy (*9*) from and to the spin system. In this context, it is interesting to ask how invar materials respond to laser-induced heating on the picosecond time scale and to determine the lattice dynamics induced by counteracting contributions of phonon and spin stresses.

One approach for the separation of the magnetic response from the ever-present phonon contribution to the lattice dynamics in laser-excited metals is to compare the structural response above and below the magnetic ordering temperature (*8*–*10*). This is sometimes prohibited by irreversible modifications of the material under heating. A demagnetized state can also be created transiently by femtosecond laser excitation (*13*, *14*) and characterized by applying a pump-probe sequence, where a second pump pulse excites the nonequilibrium state generated by the first pump pulse. Double-pulse excitation experiments not only have been used to demonstrate intriguing coherent control of the magnetization (*15*–*17*) and lattice dynamics (*18*, *19*) but they also revealed that the induced magnetization dynamics, (*20*) total transient demagnetization (*21*), and magnetic anisotropy (*22*) critically depend on the pulse-to-pulse separation.

Invar behavior is found in many Fe-containing alloys (*1*, *23*). The magnetic recording medium FePt in the fully ordered L1_0_ phase is receiving particular attention due to its large uniaxial magnetic anisotropy energy (*K*_u_ > 4.5 J/cm^3^) (*24*), which sustains nanoscopic magnetically stable bits with perpendicular magnetization. The envisioned heat-assisted magnetic recording scheme (*25*) aims at improving the magnetic information densities to exceed 2 Tb/in^2^ in commercial products of the near future (*26*). The possibility to grow magnetic, oriented nanograins with a high degree of structural order makes this material an ideal candidate for studying the lattice using time-resolved diffraction techniques. In a recent ultrafast x-ray diffraction (UXRD) study, we have found a short-lived lattice contraction along the short out-of-plane *c* axis of the tetragonal unit cell of a nanogranular FePt film on a substrate, whereas continuous epitaxial thin films merely expanded under otherwise identical excitation conditions (*27*). Previously, ultrafast electron diffraction experiments had reported a transient *c*-axis contraction and in-plane expansion for freestanding FePt nanograins (*11*). Spin-polarized density functional theory consistently predicts this tetragonal distortion when comparing the spin-ordered ferromagnetic ground state to the paramagnetic phase with full spin disorder (*11*). In the same paper, a strongly anisotropic phonon stress was predicted, seven times larger in-plane than out-of-plane (*11*). In all three cases the material is the L1_0_ phase of FePt. Therefore, the variability of the measured ultrafast dynamics suggests that the morphology and substrate-induced strain must have an important influence on the lattice dynamics at ultrafast time scales.

Here, we use fluence-dependent UXRD experiments on granular FePt thin films to show experimentally that the initial contraction originates from spin entropy, as it saturates for high fluence when the spin system is disordered. Weak excitation pulses trigger an initial contraction driven by spin stress, but expansive lattice stresses prevail after about 3 ps. The direct connection of spin disorder with the contractive stress is revealed by double-pulse excitation scenarios. When a strong first excitation pulse has essentially disordered the spin system, a second excitation pulse applied after a short delay only triggers expansion.

However, if the second pulse arrives about 100 ps later, the spin order has partially recovered, and the second pulse yields a contraction. This time scale for the recovery of the contractive stress is dictated by thermal transport and identical to the time scale of remagnetization observed in time-resolved magneto-optical Kerr effect (tr-MOKE) measurements. We model the coupled out-of-plane and in-plane lattice response of the nanograins using finite-element modeling (FEM) by varying the amplitude of the uniaxial contractive stress component $\sigma_{⊥}^{\text{sp}}$ associated with the spin disorder, which is the essential parameter for describing the two-pulse experiments. To provide a solid experimental basis for our interpretation, we compare granular films composed of FePt grains in a carbon matrix to continuous films, where the in-plane expansion on the picosecond time scale is forbidden by symmetry.

## RESULTS

### Time-resolved and static expansion

**Fig. 1:**
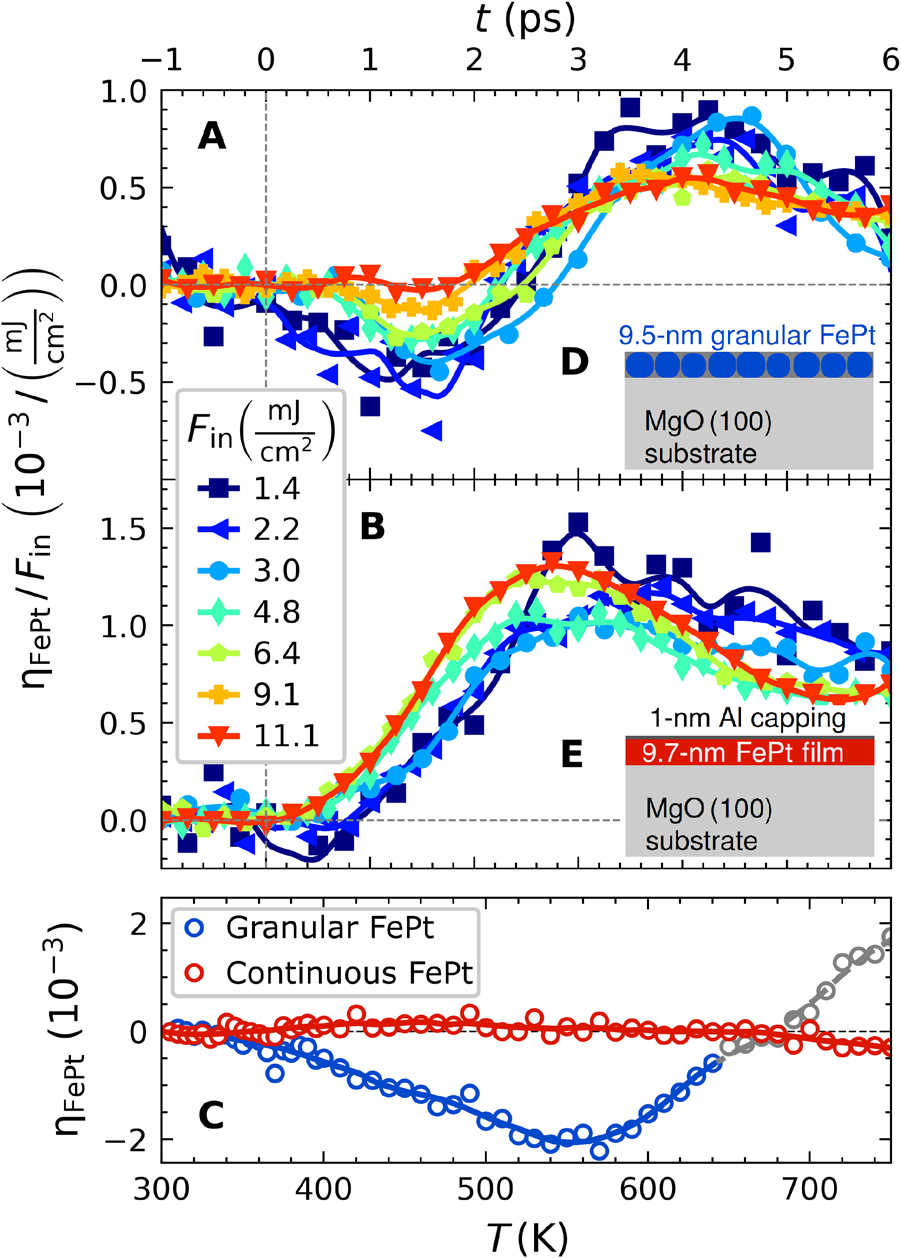
Comparison of granular and continuous FePt film responses to laser excitation and equilibrium heating. (**A**) Normalized transient out-of-plane strain η_**⊥**_ in FePt derived from the Bragg peak shift in UXRD experiments involving excitation of the granular FePt film with various incident fluences *F*_in_. The observed strain is normalized to *F*_in_. (**B**) Same for the continuous film. (**C**) Out-of-plane strain η_⊥_ upon equilibrium heating for both samples. Points above 650 K are grayed out, because the Bragg peak intensity decrease by 20% of the granular sample may indicate a slight sample degradation. Solid lines serve as guide to the eye. The insets (**D**) and (**E**) schematically depict the sample structures.

We first discuss the lattice response of FePt to laser excitation and equilibrium heating. Figure 1 (A and B) compares the lattice response of a granular and a continuous film of similar thickness to 100-fs pump laser pulses for incident laser fluences ranging from *F*_in_ = 1.4 to 11 mJ/cm^2^ (see Materials and Methods for details). To show that lattice expansion beyond 3 ps is approximately proportional to *F*_in_ and thus to the energy density, we have normalized the observed out-of-plane strain η_⊥_ to the incident laser fluence. Because the phonon system hosts most of the energy density at this time, the strain per fluence is approximately the same, and variations are due to energy absorbed in the spin system. The central finding for the granular film (Fig. 1A) is the pronounced contraction in the first 2 ps. Its absolute value is maximized for medium laser fluences, and the contraction disappears upon increasing the laser fluence further (see Supplementary Materials for the unscaled data). This already hints at the spin disorder as the driving mechanism of the contraction. The UXRD results in Fig. 1B show that the contraction is essentially absent for the continuous FePt film at all fluences. The small delay of the expansion observed in Fig. 1B for low laser fluences suggests that expansive and contractive out-of-plane stresses have different time dependences. Although the thermal expansion of bulk FePt solid solutions of different composition has been studied (*28*–*31*), static characterization of the out-of-plane expansion for continuous and granular L1_0_-ordered thin films approaching the Curie temperature *T*_C_ ≈ 700 K was, so far, not available. Our results in Fig 1C show that the out-of-plane dimension of L1_0_-FePt behaves invar-like for the continuous film and even exhibits negative thermal expansion (NTE) for the granular FePt sample. The in-plane thermal expansion coefficient of FePt matches the value 1 × 10^−5^ K^−1^ of the MgO substrate (see Supplementary Materials), so that epitaxial stresses on the thin film upon equilibrium heating are expected to be small (*11*, *29*, *31*). Figure 1 thus directly contrasts that the FePt out-of-plane strain η_⊥_ exhibits a pronounced difference between equilibrium heating, which shows NTE and invar behavior for granular and continuous FePt films, respectively, and ultrafast laser heating, where FePt mainly expands out-of-plane showing a positive strain η_⊥_ = Δ*c*/*c*. We attribute the differences in η_⊥_(*t*) for the nanogranular and continuous FePt to the different magnitudes of in-plane strain η_∥_(*t*). The probed region is almost homogeneously heated as the excitation spot is three times larger than the probe pulses. Any in-plane stresses are therefore balanced by the adjacent unit cells for the continuous FePt film. The in-plane strain propagation from the edge of the excitation region to the probed region at the sound velocity sets the 100-ns time scale (much longer than those investigated in the time-resolved experiments) on which this in-plane fixation is relieved. For the granular FePt film, the inhomogeneity at the carbon-FePt interface enables transverse stresses and strains even on picosecond time scales, whereas they are forbidden by symmetry in the continuous film case. Under static heating conditions, both the substrate and the thin film can relax in-plane, which creates additional contractive elastic stresses out-of-plane via the Poisson effect. Thus, the static out-of-plane NTE of the granular film is reduced to an invar behavior in the continuous film, for which the in-plane expansion of FePt and hence the Poisson effect are limited by the epitaxial clamping to the substrate.

### Double-pulse excitation: Spin stress tuning

**Fig. 2:**
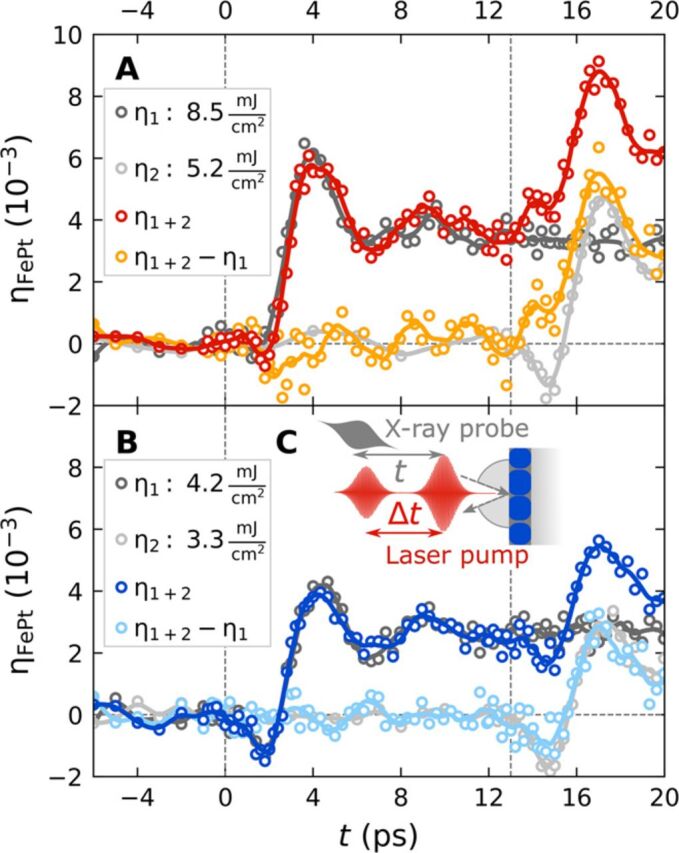
UXRD with double-pulse excitation. (**A**) Transient strain η(*t*) of the granular FePt film from UXRD with single- and double-pulse excitation. The first pulse at *t* = 0 has a fluence of *F*_in,1_ = 8.5 mJ/cm^2^, and the second pulse at 13 ps is weaker (*F*_in,2_ = 5.2 mJ/cm^2^). The nonequilibrium strains η_ne_(*t*) = η_1 + 2_(*t*) − η_1_(*t*) (orange) are derived by subtracting the dark gray curve from the red curve. This strain is induced by the photoexcitation in the nonequilibrium conditions set by the first pulse. (**B**) Same for weaker pump pulses *F*_in,1_ = 4.2 mJ/cm^2^ and *F*_in,2_ = 3.3 mJ/cm^2^, which only partially demagnetize the film. (**C**) Relative timing of the double-pulse excitation experiments.

The results of a double-pulse excitation scheme displayed in Fig. 2A confirm that the spin excitations drive the contraction in the granular FePt film. In these experiments, we use a first strong laser pulse (p_1_) to saturate the spin excitations and a second, weaker laser pulse (p_2_) for triggering subsequent dynamics with a delay Δ*t*. The ultrashort x-ray probe pulse detects the lattice dynamics η_⊥_(*t*) that is induced by this double-pulse excitation, as schematically depicted in Fig 2C. The dark gray data from Fig. 2A show the UXRD signal that characterizes the strain η_1_(*t*) due to a single strong pulse with *F*_in,1_ = 8.5 mJ/cm^2^ at *t* = 0, which almost exclusively leads to expansion of the FePt film. In contrast, the light gray data representing the strain η_2_(*t*) generated by a weaker single pulse with *F*_in,2_ = 5.2 mJ/cm^2^ arriving at ∆*t* = 13 ps show a pronounced contraction at the onset of the second pulse, consistent with the fluence series in Fig. 1A. When both pulses excite the sample with the delay set to ∆*t* = 13 ps, we observe the strain η_1 + 2_(*t*) (red data points). The orange points represent the additional strain η_ne_(*t*) = η_1 + 2_(*t*) − η_1_(*t*), which is induced by the photoexcitation of the sample in the nonequilibrium conditions previously set by the first pulse. It mainly differs from η_2_(t) (light gray curve in Fig. 2A) in the first 2 ps after the second laser pulse arrives. Clearly, there is no contraction at *t* = 15 ps just after the second pulse, if the sample was pre-excited with the first pulse. We conclude that the first pulse has essentially saturated the spin excitations. Figure 2B confirms this interpretation by reducing the fluences to *F*_in,1_ = 4.2 mJ/cm^2^ and *F*_in,2_ = 3.3 mJ/cm^2^ with the same timing. Now, η_ne_(*t*) shows approximately half of the contraction at *t* = 15 ps compared to η_2_(*t*) (light gray data) because the first pulse does not fully saturate the spin excitations.

**Fig. 3:**
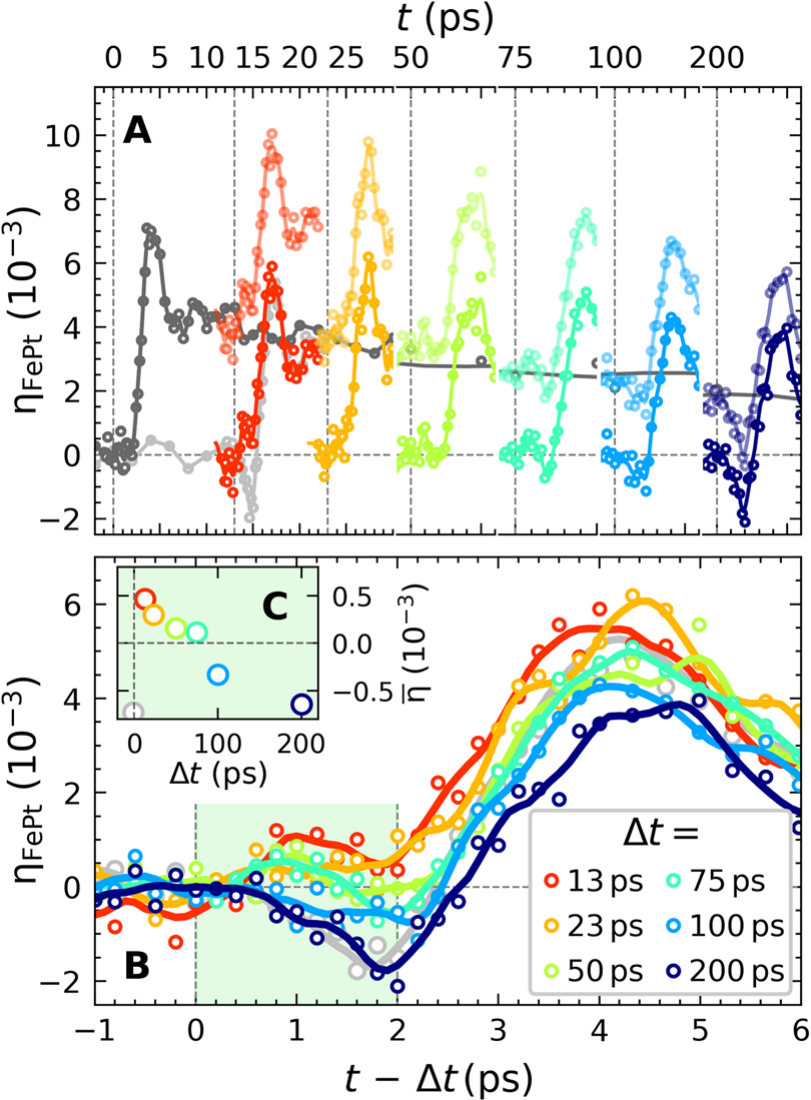
Recovery of the spin entropy–driven lattice contraction. (**A**) Dark gray: transient strain η_1_(*t*) from UXRD with single-pulse excitation at *t* = 0 (*F*_in,1_ = 8.5 mJ/cm^2^) for the granular film. Light-colored data: strain η_1 + 2_(*t*) observed for double-pulse excitation with the same first pulse and a second pulse with fluence (*F*_in,2_ = 5.2 mJ/cm^2^) after Δ*t* = 13, 23, 50, 75, 100, and 200 ps. The bright-colored data represent η_ne_(*t*) = η_1 + 2_(*t*) − η_1_(*t*). (**B**) Comparison of η_ne_(*t* − Δ*t*) from (A) to the strain η_2_(*t*) obtained for excitation only with the second pulse (gray). (**C**) Average strain η_ne_(*t* − Δ*t*) within the first 2 ps.

Our double-pulse scheme also allows monitoring the recovery of the contractive stress by adjusting the timing between the excitation pulses for constant *F*_in,1_ and *F*_in,2_. Figure 3A depicts the results from Fig. 2A for tuning the double-pulse time delay Δ*t*. Again, the gray line shows the strain induced only by the first pump pulse with *F*_in,1_ = 8.5 mJ/cm². Within 200 ps, the cooling of the FePt lattice reduces the transient strain from η_⊥_ = 4 × 10^−3^ to the half value. The light-colored lines show the strain η_1 + 2_(*t*) observed for double-pulse excitation, and the bright colors show the nonequilibrium strain η_ne_(*t*) = η_1 + 2_(*t*) − η_1_(*t*). Figure 3B reproduces these data on a time axis where *t* = 0 indicates the arrival of the second pulse and compares η_ne_(*t* − Δ*t*) to η_2_(t) (light gray), i.e., the response to the second pulse with and without pre-excitation. For a pulse delay of Δ*t* = 200 ps, η_ne_(*t* − Δ*t*) and η_2_(t) nearly coincide in the first 3 ps, indicating a reordering of the spin system within this time scale. For time delays shorter than Δ*t* = 75 ps, the lattice expansion prevails. The red line (Δ*t* = 13 ps) transforms continuously into the dark blue line (Δ*t* = 200 ps) with increasing time delay, indicating the emergence of the contractive stress as the spin system can be disordered again by the second pulse.

### Magnetization dynamics and energy density

To corroborate our findings about the spin stress contribution to the lattice dynamics, we analyze the magnetic system. The spin stress contribution to the strain response must vanish if the magnetic system is in a state close to its maximal entropy that can be reached either thermally or via laser-induced demagnetization. According to recent FEM simulations of the field enhancement effects in the optical absorption of a similar nanogranular sample (*32*), the temperature change due to the inhomogeneous optical absorption of the irregularly shaped FePt nano-islands varies between 10 and 30%. For *F*_in,1_ = 8.5 mJ/cm², we therefore estimate the temperature rise to be in the range Δ*T* = 300 to 700 K. The majority of the nanogranular FePt will be transiently heated above the Curie temperature, which is about *T*_C_ ≈ 650 to 700 K for the current particle size (*33*).

**Fig. 4:**
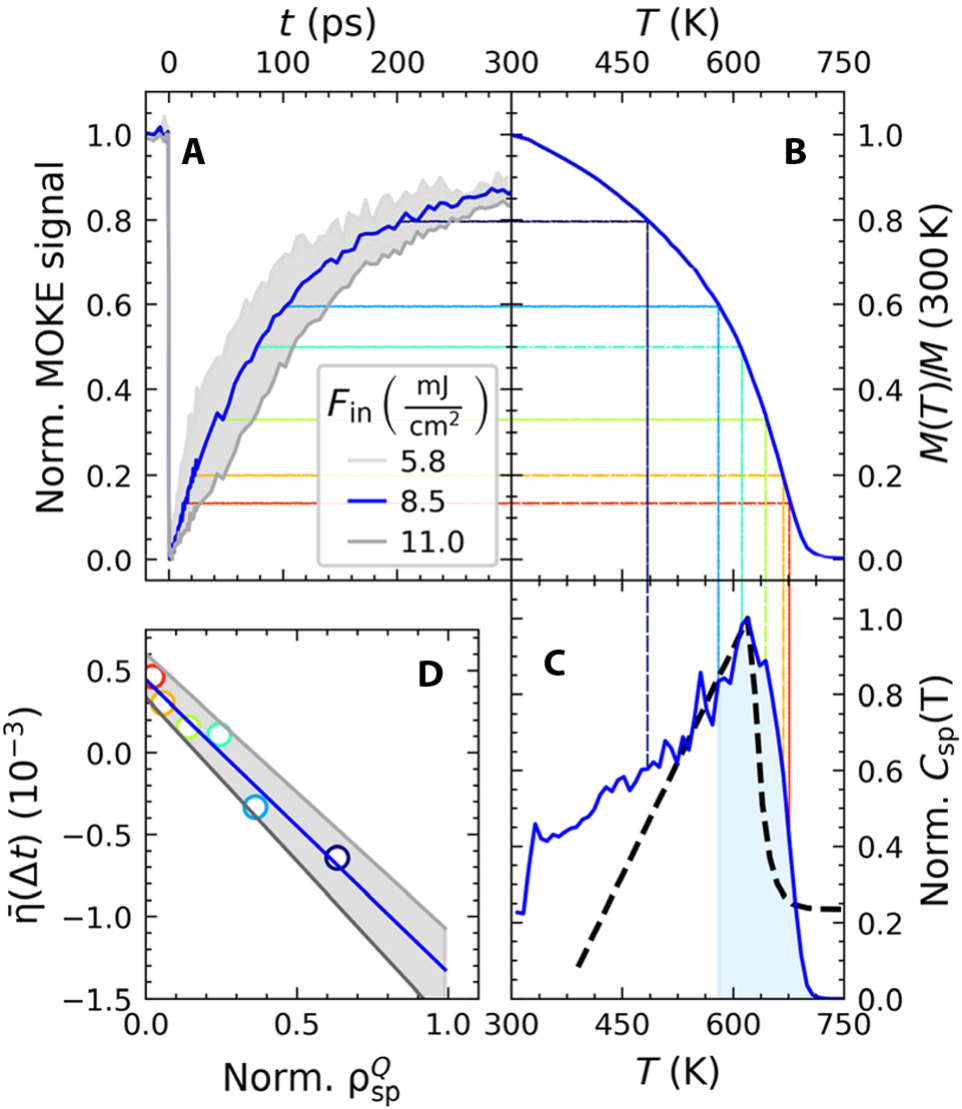
Semiquantitative approximation of the remaining spin energy from tr-MOKE measurements. (**A**) Transient MOKE data for the granular FePt film for various fluences, normalized (Norm.) to their maximum demagnetization. (**B**) Equilibrium magnetization *M*(*T*) measurement for a similarly prepared granular FePt film obtained by vibrating sample magnetometry (VSM). (**C**) Spin-specific heat as derived via $C_{\text{sp}}\approx M\frac{\partial M}{\partial T}$ (blue line) from *M*(*T*) and calculations (*36*) (thick black dashed line). The colored dashed lines connect the graphs at selected times for which partial recovery of the spin entropy–driven contraction is observed (compare Fig 3). (**D**) Average strain $\bar{\eta }(\Delta t)$ in the first 2 ps after the second pulse (green area in Fig. 3B) as a function of the energy density $\Delta \rho_{\text{sp}}^{Q}(\Delta t)$ that this second pulse can still introduce into the spin system. The light blue shaded area in (C) visualizes $\Delta \rho_{\text{sp}}^{Q}$ for the case of *T*(100 ps) = 580 K, which gives rise to the light blue circle in (D).

Figure 4A contains tr-MOKE data for three selected fluences. We assume nearly full demagnetization for the incident fluence of 11 mJ/cm², as the signal does not increase beyond this fluence. Consistent with literature, this sets the initial demagnetization for the pulses (8.5 mJ/cm²) used in the UXRD experiment from Figs. 3A and 2A at 85%. Because of the large out-of-plane anisotropy and the single-domain character of the grains (no domain wall propagation), we can use the tr-MOKE signal recorded with an external field of ±0.7 *T* as an estimation of the time-dependent average magnetization *M*(*t*) of the sample (*34*). The static magnetization curve *M*(*T*) of the granular FePt sample is depicted in Fig 4B. To relate the UXRD and tr-MOKE signal, we calculate an estimate for the spin contribution to the heat capacity (Fig. 4C) according to the mean field theory relation (*35*) $C_{\text{sp}}\propto \frac{\partial M^{2}}{\partial T}=M\frac{\partial M}{\partial T}$, which agrees reasonably well with recent theoretical predictions represented by the dashed line (*36*).

The colored dashed lines in Fig. 4 (A to C) indicate how the MOKE signal, which is proportional to *M*(*t*), is related to the auxiliary temperature *T*(*t*) of the spin system for the specific time *t* = Δ*t* of the UXRD experiment. This temperature is used to estimate the energy density $\Delta \rho_{\text{sp}}^{Q}=\int_{T}^{\infty }C_{\text{sp}}(T\prime )dT\prime$, which would be required to fully saturate the heat capacity *C*_sp_(*T*) of the spin system. In a first-order analysis, the individual stresses from electrons, phonons, and spins $\sigma_{e,\text{ph},\text{sp}}=\Gamma_{e,\text{ph},\text{sp}}\rho_{e,\text{ph},\text{sp}}^{Q}$ are directly proportional to the heat energy densities $\rho_{e,\text{ph},\text{sp}}^{Q}$, and the dimensionless macroscopic Grueneisen coefficients Γ_*e*, ph, sp_ describe the efficiency for generating stress from energy in each of the three systems (*8*).

To combine UXRD and tr-MOKE, we reproduce on the vertical axis of Fig. 4D the average lattice strain $\bar{\eta }$ from Fig. 3C, while the horizontal axis quantifies the fraction of the energy density $\Delta \rho_{\text{sp}}^{Q}(\Delta t)$ that the second pulse can still introduce into the spin system according to (Fig. 4C). For simplicity, we assume that after a short time delay Δ*t* = 15 ps, the FePt is still nearly fully demagnetized (Fig. 4A), and almost no energy density $\Delta \rho_{\text{sp}}^{Q}$ can be deposited into the spin system. Hence, the second pulse only induces expansion, i.e., positive $\bar{\eta }(\Delta t)$ in Fig. 4D by exciting electrons and phonons. With increasing Δ*t*, the contractive stress $\sigma_{\text{sp}}=\Gamma_{\text{sp}}\Delta \rho_{\text{sp}}^{Q}$ increases. The slope of Fig. 4D is proportional to the macroscopic Grueneisen constant Γ_sp_ of the spin system, which must, in fact, be negative to support the observed NTE or invar behavior (*3*, *37*).

### Modeling

We experimentally find that the spin stress contribution recovers on a 100-ps time scale, consistent with the remagnetization of the grains. As domain wall propagation is not relevant within the nanoscopic grains on this time scale, the dynamics are governed by thermal transport to the carbon matrix and the substrate. Figure 4C illustrates that the energy density $\rho_{\text{sp}}^{Q}$ and the associated spin entropy density $S_{\text{sp}}\propto \rho_{\text{sp}}^{Q}/T$ that can be induced by a second excitation after a given time delay Δ*t* are finite. Statistical mechanics limits the maximum spin entropy to *S*_sp_ = *Nk*_B_ ln (2*J* + 1), where *J* is the angular momentum per magnetic atom. This saturation provides the necessary mechanism for the reduced contractive stress contribution at high fluences in the otherwise linear stress-strain relations. NTE generally requires an increasing entropy with decreasing volume (*23*).

The main features of the fluence-dependent responses observed for the continuous and granular films (Fig. 1) and of the two-pulse excitation experiments (Figs. 2 and 3) can be qualitatively understood in the light of a simplified equation of motion [see Materials and Methods for the full three-dimensional (3D) equation]

$$\rho \frac{\partial^{2}u_{⊥}}{\partial t^{2}}=\frac{\partial }{\partial z}(\underset{\text{elast}.\sigma_{⊥}}{\underbrace{C_{33}\frac{\partial u_{⊥}}{\partial z}}}+\underset{\text{elast}.\sigma_{⊥}^{\text{Poi}}}{\underbrace{2C_{31}\eta_{∥}}}-\underset{<0}{\underbrace{\sigma_{⊥}^{\text{sp}}}}-\underset{>0}{\underbrace{\sigma_{⊥}^{e-\text{ph}}}})$$ (1)

At equilibrium, negative strain $\eta_{⊥}=\frac{\partial u_{⊥}}{\partial z}<0$ occurs only if a contractive spin stress $\sigma_{⊥}^{\text{sp}}$ and the elastic Poisson stress contribution $\sigma_{⊥}^{\text{Poi}}(t)\sim \eta_{∥}(t)$ induced by in-plane strain η_∥_ add constructively to overcome the expansive out-of-plane stress $\sigma_{⊥}^{e-\text{ph}}$ imposed by hot electrons and phonons. Equation 1 is valid in the case of a thin FePt needle, i.e., a cylinder with radius much smaller than height (*r* ≪ *d*), because this allows us to assume that in-plane strains η_∥_ are relaxed and equal in *x* and *y* directions. The main reason for writing Eq. 1 is to see that it can be further simplified for the continuous film because $\sigma_{⊥}^{\text{Poi}}$ is absent at ultrafast time scales for which η_∥_ = 0 for symmetry reasons. Thus, on ultrafast time scales, Eq. 1 with $\sigma_{⊥}^{\text{Poi}}=0$ is exact, and the Poisson stress makes the out-of-plane response of the granular film response substantially different.

**Fig. 5:**
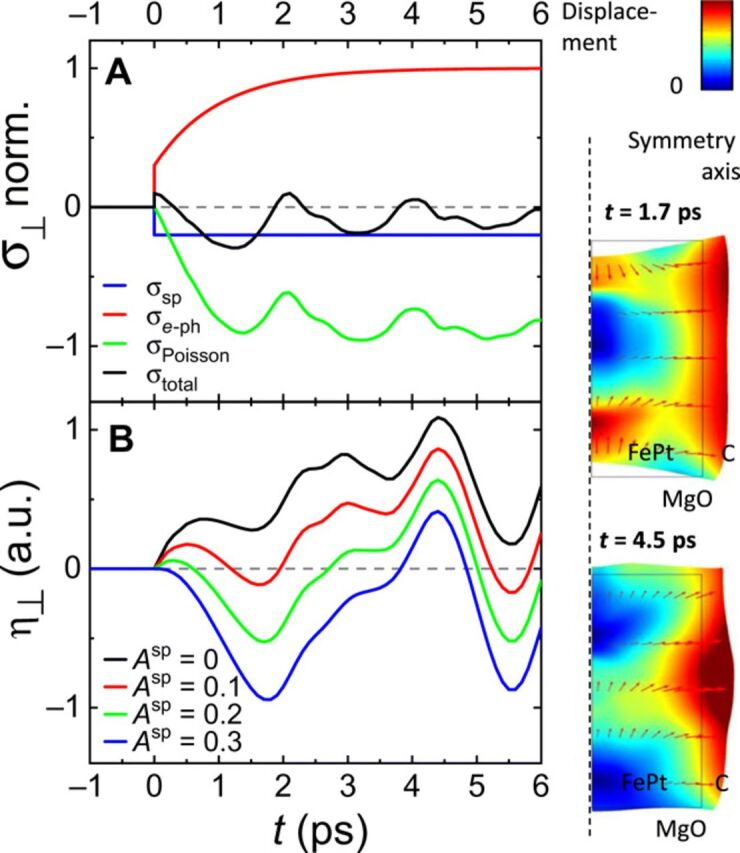
FEM modeling of the mechanical response of a granular FePt film. (**A**) Time-dependent out-of-plane stresses σ_⊥_(*t*) acting on FePt for *A*^sp^ = 0 (no spin stress, solid lines) and *A*^sp^ = 0.2 (dashed lines). The Poisson stress component was deduced from the computed in-plane strains. (**B**) Average out-of-plane strain η_⊥_(*t*) in arbitrary units (a.u.) computed for various ratios *A*^sp^ of the spin and electron-phonon stress amplitudes. The computed FePt deformation at instants corresponding to maximal initial contraction and expansion for *A*^sp^ = 0.2 are illustrated at the right.

We complement this simple analysis of the FePt deformation dynamics by FEM simulations using the actual FePt nanostructure dimensions and considering 3D, nonsimplified equations of motion (see Materials and Methods and Supplementary Materials for technical details). The results for the granular FePt film are shown in Fig. 5, while those obtained for continuous films and free grains can be found in the Supplementary Materials. In each case, various values of the relative amplitude *A*^sp^ of the spin stress contribution were used to mimic its variation in the context of fluence dependence (Fig. 1—saturation of the contraction) and two-pulse excitation (Figs. 2 and 3—time-dependent recovery of the spin stress). A complete reproduction of the measured time-resolved signals is challenging, as it would require to precisely take into account the morphological dispersion of the FePt grains and the heat transfer to the carbon. Nevertheless, enable a good qualitative reproduction of the strain dynamics measured for granular FePt films, as can be seen by comparing the measured strain (Figs. 1A and 3B) with the simulated strain in Fig. 5B.

Figure 5A illustrates the electron-phonon, spin, and Poisson stress contributions, which drive the out-of-plane strain dynamics of granular films (Eq. 1). As expected, in the absence of the contractive spin stress (*A*^sp^ = 0), the total stress almost always remains positive. The computed out-of-plane strain dynamics correspond to oscillations of the FePt nanostructure configuration around an expanded equilibrium, with a total absence of out-of-plane compression throughout the motion (Fig. 5B). These computed strain dynamics agree well with experimental strain data where the spin contribution is strongly reduced via the use of high-fluence light pulses (Figs. 1 to 3). Conversely, a sufficient amplitude of *A*^sp^ (e.g., *A*^sp^ = 0.2, as in Fig. 5A) creates a negative average value of the computed total stress enabling out-of-plane FePt contractions (Fig. 5B), in agreement with the experimental observation of a contraction at the beginning of the dynamics. Moreover, the computed strains shown in Fig. 5B qualitatively reproduce both the maximal contraction at 1.7 ps and maximal expansion at 4.5 ps, observed in the context of both low-fluence single-pump experiments (Fig. 1A) and double-pulse excitation with large delay between pump pulses, i.e., recovered spin order (Fig. 3B). The FePt deformation computed at these two instants is illustrated for *A*^sp^ = 0.2 in Fig. 5. For the same range of *A*^sp^, our FEM modeling of a continuous film (see Supplementary Materials) reproduces the absence of a contraction and the 0.5-ps phase shift observed in Fig. 1B for increasing fluence. Conversely, the Poisson effect is enhanced for free FePt grains (see Supplementary Materials) because the in-plane displacement is unconstrained. Our simulations qualitatively reproduce the large out-of-plane contractions observed previously (*11*).

A systematic variation of the simulation parameters shows that a reasonable agreement with the experimental data can be obtained only by assuming an anisotropic electron-phonon stress, with nonequal out-of-plane and in-plane amplitudes $\sigma_{⊥}^{e-\text{ph},0}=A^{\text{ani}}\;\sigma_{∥}^{e-\text{ph},0}$. However, the optimal value of the anisotropy parameter *A*^ani^ ≈ 3 used for the simulations shown in Fig. 5 is more than twice smaller than predicted (*11*). Including an in-plane expansion resulting from spin stress (*11*) would even reduce *A*^ani^ further. Although the overall agreement between simulations and experiments is good, the simulations systematically underestimate the expansion beyond 3 ps observed experimentally. This may be compensated for by adding expansive stress in the carbon shell resulting from heat transfer from the FePt, which would decrease the Poisson stress $\sigma_{⊥}^{\text{Poi}}$ acting on FePt on long time scales.

Our modeling shows that the difference in the response of free grains, a granular film, and the continuous film mainly originates from the different in-plane boundary conditions, which suppress or partially allow the Poisson effect. We can reproduce the essential conclusion drawn from the double-pulse experiment (Fig. 3) that the initial out-of-plane contraction is driven by spin stress.

## CONCLUSION

In conclusion, we have shown that laser-generated spin entropy drives a pronounced but short-lived lattice contraction of nanogranular FePt films in the L1_0_ phase. In a double-pulse excitation scenario, the absence of a contraction after the second laser pulse quantifies the contractive stress contribution of the spin excitations, as they saturate when the FePt temperature approaches *T*_C_. Fluence-dependent transient MOKE data confirm that the relaxation of the magnetization occurs on the same time scale as the spin entropy–driven contraction reappears.

Our elastic continuum modeling clarifies the important role of the Poisson effect in establishing the transient contraction of the granular film, which is not observed for the continuous film. We are confident that this double-pulse excitation scenario can be developed into a versatile tool for investigating coupled systems with many degrees of freedom, when a phase transition leads to the saturation for one of the driving stresses of the lattice response.

## MATERIALS AND METHODS

### X-ray and MOKE experiments

We performed laser-based UXRD pump-probe experiments with an x-ray pulse duration (*38*) of approximately 200 fs on two FePt thin films in the ordered L1_0_ phase grown on MgO (001) oriented substrates. We observe the time-dependent evolution of the (002) FePt diffraction peak, from which we deduce the time-resolved out-of-plane lattice strain of the FePt layer η_⊥_(*t*). The samples are excited by p-polarized pump pulses with a duration of 100 fs at a central wavelength of 800 nm, which are incident under 45° relative to the surface normal. The laser spot size of approximately 1.6 mm by 1.3 mm (full width at half maximum) ensures that a homogeneously excited sample area is probed by the x-rays that have rhombical 0.3 mm–by–0.3 mm profile (*39*). The tr-MOKE setup (*34*) uses comparable excitation parameters. For experimental details, see Supplementary Materials. Static x-ray diffraction measurements at different sample temperatures were recorded using a commercial diffraction setup (Rigaku SmartLab 9 kW system).

### Sample preparation

A continuous FePt thin film was prepared by magnetron-sputtering Fe and Pt from a composite FePt target onto a substrate preheated to 500°C. Similarly, a granular FePt film was prepared at a slightly higher substrate temperature of 650°C by adding approximately 30 volume % of carbon to the sputtering target. X-ray reflectivity measured the sample thicknesses to be about *d* = 9.5 nm, where the continuous film is covered with an additional 1-nm layer of oxidized Al (*27*). According to scanning electron microscopy images of similarly prepared samples (see Supplementary Materials), the size distribution of the FePt nanograins segregated in a carbon matrix within the granular film is centered at approximately 8 nm. This nano-morphology yields a very large coercive field of approximately μ_0_*H* = 5 T, whereas the coercive field of the continuous film μ_0_*H* = 0.4 T is substantially smaller because of the possibility of domain wall motion that cannot occur in the nanogranular samples (*40*). The sample structures are schematically depicted as insets (C) and (D) in Fig. 1, and their properties and the measurement technique have been described in a previous publication (*27*).

### FEM modeling

Finite-element simulations were performed using the Structural Mechanics Module of the COMSOL commercial software. It determines the spatiotemporal variations of displacement *u*_i_(*x*_1_, *x*_2_, *x*_3_, *t*) by the numerical, approximation-free resolution of the continuum mechanics equation of motion in all Cartesian directions *x*_i_

$$\rho \frac{\partial^{2}u_{i}}{\partial t^{2}}=\sum_{j=1}^{3}\frac{\partial }{\partial x_{j}}(\sum_{k,l=1}^{3}C_{\text{ijkl}}\eta_{\mathrm{kl}}-\sigma_{\mathrm{ij}}^{\text{ext}})\text{with the strain}\;\eta_{\mathrm{kl}}=\frac{1}{2}(\frac{\partial u_{k}}{\partial x_{l}}+\frac{\partial u_{l}}{\partial x_{k}})$$

The simulation system was composed of a FePt cylinder with the radius *r* = 4 nm and *d*=10 nm height encapsulated by a cylindrical carbon shell of 2-nm thickness and same height supported on a MgO substrate. Note that the choice of such an axially symmetric geometry allowed us to perform 2D simulations, which are computationally much less expensive than 3D ones. Perfect mechanical contact was assumed at all internal interfaces of the system. Vanishing in-plane displacement was imposed on the lateral surface of the simulation domain, 6 nm away from its symmetry axis, to describe the absence of lateral contraction in films. Stress-free and low-reflecting boundary conditions were, respectively, used at the top of the FePt–carbon film and at the bottom of the MgO substrate.

FePt elastic anisotropy was neglected, and all materials were described by their density ρ, Young modulus *Y*, and Poisson ratio ν as listed in Table 1.

**Table 1:** Elastic parameters used in the modeling.

	**FePt**	**C**	**MgO**
ρ (kg/m^3^)	14,700	2000	3580
*Y* (GPa)	237	200	249
ν	0.31	0.2	0.18

The time-dependent displacement fields *u*_i_(*t*) induced in this system by its sudden excitation were computed in the time domain, and the average out-of-plane strain in FePt $\eta_{⊥}(t)=\frac{\partial u_{z}(t)}{\partial z}$ was deduced by spatial integration.

The laser-induced excitation was described by a time-dependent diagonal matrix obtained by summing the contributions of an expansive, anisotropic electron-phonon stress with components $\sigma_{\mathrm{xx}}^{e-\text{ph}}(t)=\sigma_{\mathrm{yy}}^{e-\text{ph}}(t)=A^{\text{ani}}\sigma_{\mathrm{zz}}^{e-\text{ph}}(t)=A^{\text{ani}}\sigma^{e-\text{pho},0}\;(1+(\frac{\gamma_{e}}{\gamma_{\text{ph}}}-1)e^{-\frac{t}{\tau }})\Theta (t)$ accounting for energy dissipation from electrons to phonons after selective excitation of the former by light (*41*) and an instantaneously rising contractive uniaxial spin stress $\sigma_{\mathrm{zz}}^{\text{sp}}(t)=\sigma_{⊥}^{\text{sp},0}\Theta (t)$, where Θ is the Heaviside function. A τ = 1 ps electron-phonon coupling time and a $\frac{\gamma_{e}}{\gamma_{\text{ph}}}=0.3$ ratio of electron and phonon Grüneisen constants were used in the modeling. We approximate both the contractive spin stress and the expansive electron stress as instantaneous, i.e., much shorter than the 200 fs time resolution of our UXRD experiment. This is consistent with recent ultrafast electron calorimetry, which has shown that the energy transfer to the spin system in nickel is effective within the first 20 fs (*42*).

## Supplementary Material

aba1142_SM.pdf

## SUPPLEMENTARY MATERIALS

Supplementary material for this article is available at http://advances.sciencemag.org/cgi/content/full/6/28/eaba1142/DC1
