# Supplementary material for: Spin stress contribution to the lattice dynamics of FePt
Source: Sci Adv. 2020 Jul 8;6(28):eaba1142. doi: 10.1126/sciadv.aba1142 (PMC7343378; doi:10.1126/sciadv.aba1142)
Supplement: aba1142_SM.pdf [file aba1142_SM.pdf]

Science Advances
AAAS

advances.sciencemag.org/cgi/content/full/6/28/eaba1142/DC1

Supplementary Materials for

Spin stress contribution to the lattice dynamics of FePt

A. von Reppert, L. Willig, J.-E. Pudell, S. P. Zeuschner, G. Sellge, F. Ganss, O. Hellwig, J. A. Arregi, V. Uhlíř, A. Crut, M. Bargheer*

*Corresponding author. Email: bargheer@uni-potsdam.de

Published 8 July 2020, *Sci. Adv.* **6**, eaba1142 (2020)
DOI: 10.1126/sciadv.aba1142

This PDF file includes:

Sections S1 to S5
Figs. S1 to S6
References

1) Excitation fluence dependent strain of granular and continuous FePt

The fluence series of the time-resolved X-ray diffraction using single pulse excitation for both the granular and the continuous FePt film is displayed in figure S1: Figure S1 a) and b) show that the expansion maximum increases with increasing fluence. The contractive strain that occurs within the first 2ps that is observed in the granular film saturates at a contraction of approximately $2 \cdot 10^{-3}$. This threshold behavior already indicates a magnetic origin of the driving stress, as saturation of the spin contribution to the stress is predicted for a full demagnetization. The bottom panels c) and d) display the data normalized to the incident fluence as presented in the main text.

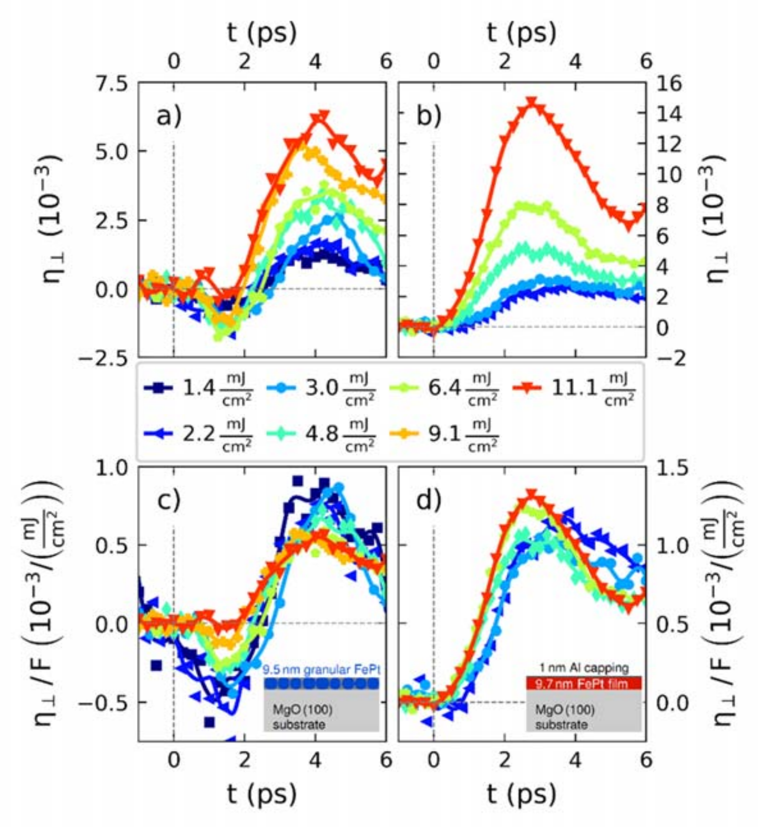

Figure S1: Comparison of the fluence dependent lattice dynamics of the two FePt thin films after laser excitation. a) and b) display the time dependent strain for the granular and continuous FePt film respectively. The data normalized to fluence are displayed in the low panel c) and d) below, where the insets display the schematic sample structure.

2) Temperature dependent thermal expansion of the granular and continuous FePt thin film

The static diffraction curve as well as the temperature dependent experiments on the FePt thin films that have been conducted on the samples after the time-resolved experiments were finished are depicted in figure S2. The static diffraction experiments were carried out at a Rigaku 9kW SmartLab system using a 4-circle goniometer that uses the characteristic Cu-$K_{\alpha 1}$ radiation (λ ($K_{\alpha 1}$) = 1.54 Å) of a rotating anode X-ray tube for diffraction. The sample was kept in an inert gas atmosphere inside a carbon dome chamber, while it was heated up to 800K. The diffraction curve in Fig S2a) exhibits sharp and intense MgO substrate peaks and smaller diffraction peaks, which can be attributed to

the FePt thin films. In order to extract the thermal expansion strain $\eta_{\perp,eq}$ of the FePt thin films and the MgO substrate, we observed the FePt (002) and the MgO (004) shift as a function of the sample temperature. From similar experiments we extract the in-plane strain upon equilibrium heating $\eta_{||,eq}$ by monitoring the peak shift of the FePt (220) and the MgO (440) diffraction peak.

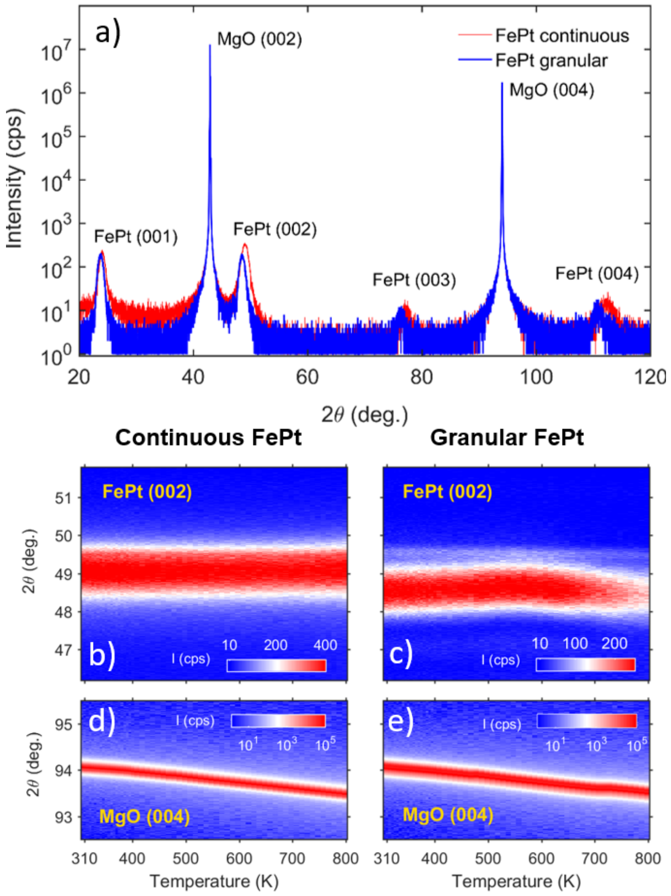

Figure S2 Static X-ray diffraction results: a) Rocking curves of the granular and continuous FePt thin films. The temperature dependence for the FePt (002) reflection for the continuous (b)) and the granular(c)) FePt exhibit only a very small shift, whereas the MgO substrates in c) and d) shift considerably to lower diffraction angles. The intensity decrease for the granular FePt above 650K indicates a modification of the structure, which prohibits reliable time-resolved experiments at temperatures above T_c

The out-of-plane ($\eta_{\perp,eq}$) and in-plane strains ($\eta_{||,eq}$) that we obtain from the peak shifts by peak fits to the temperature dependent diffraction data under equilibrium heating are displayed in figure S3. Figure S3a) quantifies that the continuous film exhibits invar behavior ($\alpha_{\perp,\mathrm{FePt,cont}} \approx 0$) along the out-of-plane direction from 300K – 700K whereas the granular FePt exhibits a negative thermal expansion with an expansion coefficient of $\alpha_{\perp,\mathrm{FePt,gran,1}} \approx -9 \cdot 10^{-6}\,\frac{1}{\mathrm{K}}$ between 300K and 550K, which switches to a large positive thermal expansion of $\alpha_{\perp,\mathrm{FePt,gran,2}} \approx 23 \cdot 10^{-6}\,\frac{1}{\mathrm{K}}$ between 580 and 650K. Qualitatively similar observations have been made in the magnetovolume effect of FePt alloys with different composition by Sumiyama et al.[28]. Above 650 K we observe a decrease of the X-ray diffraction peak intensity of the granular FePt film that can be seen in Fig S2c). This

could be due to an irreversible carbon interdiffusion into the sample structure as the peak intensity remains reduced by 15% even after the sample has cooled to room temperature. This illustrates that experiments at elevated temperatures above $T > T_c \approx 700$ K modify the sample whereas transient heating above T_c on a picosecond timescale is reversible since no change of the Bragg peak intensity was observed after laser pulse excitation. The thermal expansion coefficient extracted from the MgO (004) peak shift is approximately $\alpha_{\perp,MgO} \approx 9.3 \cdot 10^{-6} \frac{1}{K}$ in reasonable agreement with literature.[31] The in-plane thermal expansion coefficient of both FePt morphologies that can be extracted from Figure S3 b) approximately matches the thermal expansion of the MgO substrate $\alpha_{\parallel,FePt} \approx 9 \cdot 10^{-6} \frac{1}{K}$.

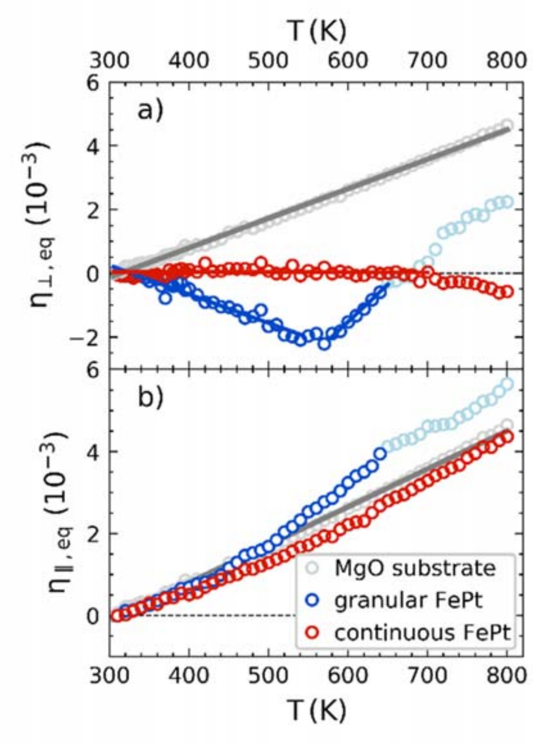

Figure S3 Static thermal expansion of the granular and continuous FePt thin film: a) Out-of-plane and b) in-plane strain extracted from the T-dependent peak shifts under equilibrium heating, where the solid lines indicate linear fits for the thermal expansion coefficients. The peak center position for a) the FePt (002) / MgO (004) and b) the FePt (220) Bragg peak were obtained by a Gaussian fits to the data from Figure S2.

3) SEM images of similarly prepared FePt samples

Figure S4 shows a scanning electron micrograph (SEM) image of a similarly prepared granular FePt sample as the one employed in our study. The bright parts are the FePt grains whereas the black parts correspond to the amorphous carbon. The images illustrate the approximate size distribution, which is centered around 8nm islands with an approximate 2nm carbon separation.

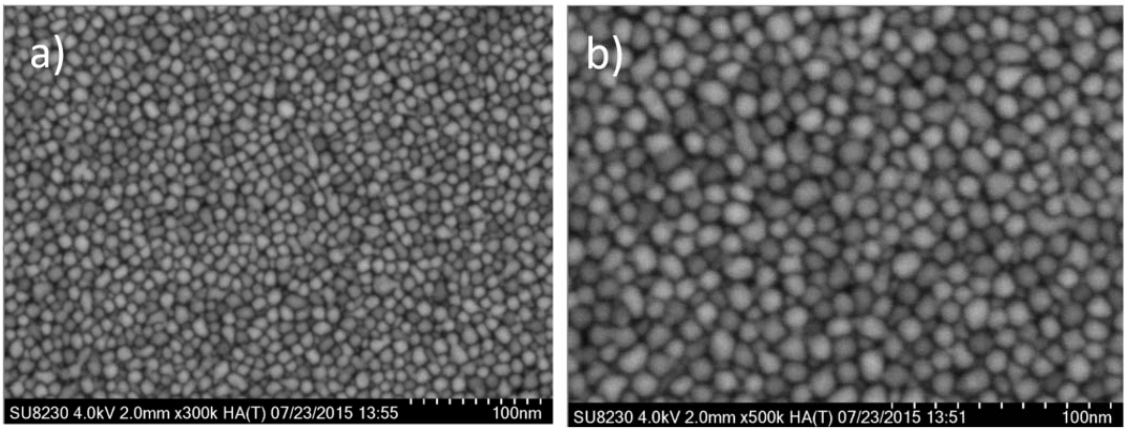

Figure S4: Scanning Electron Microscope images of similarly prepared FePt samples, that illustrate the approximate size distribution for the granular specimen studied in the main text. Bright areas correspond to the FePt islands with an average diameter of approximately 8nm. The dark spacing in between the grains is due to the carbon nanolayer, which has an average thickness on the order of 2nm.

4) FEM modeling

FEM simulations were performed for both granular and continuous FePt films. To facilitate the comparison with the literature results reported for granular, matrix-free FePt films deposited on a TEM grid, the ideal case of a free FePt grain was also considered. Figure S5 shows the geometry of the FEM simulations, indicating the boundary conditions used in each case. Vanishing in-plane displacement at the cylinder lateral surface was assumed for continuous films bound to a substrate, while in-plane motion of the FePt surface was allowed in the granular film (at the FePt/C interface) and free nanoparticle cases.

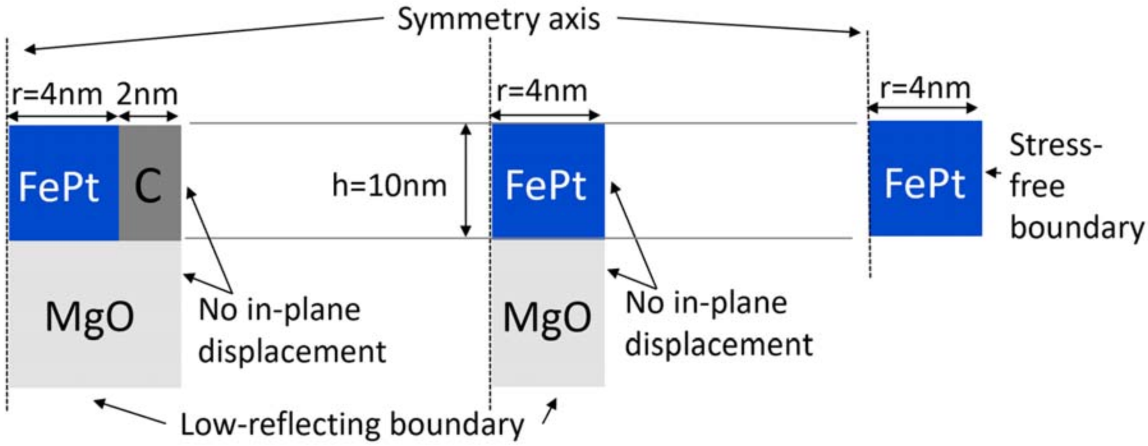

Figure S5: Geometry of the FEM simulations performed for three different FePt nanosystems: granular film (left), continuous film (middle) and free grain (right).

Fig. S6 shows the results of the FEM simulations for the continuous film (Fig. S6 a+b) and for a free FePt grain (Fig. S6 c+d), which complement those obtained for the granular film presented in Fig. 5 of the main text of the paper.
The simulations performed for the continuous film noticeably show a variation (by about 0.5 ps) with the amplitude of the spin stress contribution of the time at which the strain is maximal (Fig. S6 b), which is in agreement with experimental observations (Fig. 1b and S1b). The FEM results obtained for free grains (Fig. S6 d) are in qualitative agreement with the observations reported in the context of ultrafast electron-diffraction experiments[11].

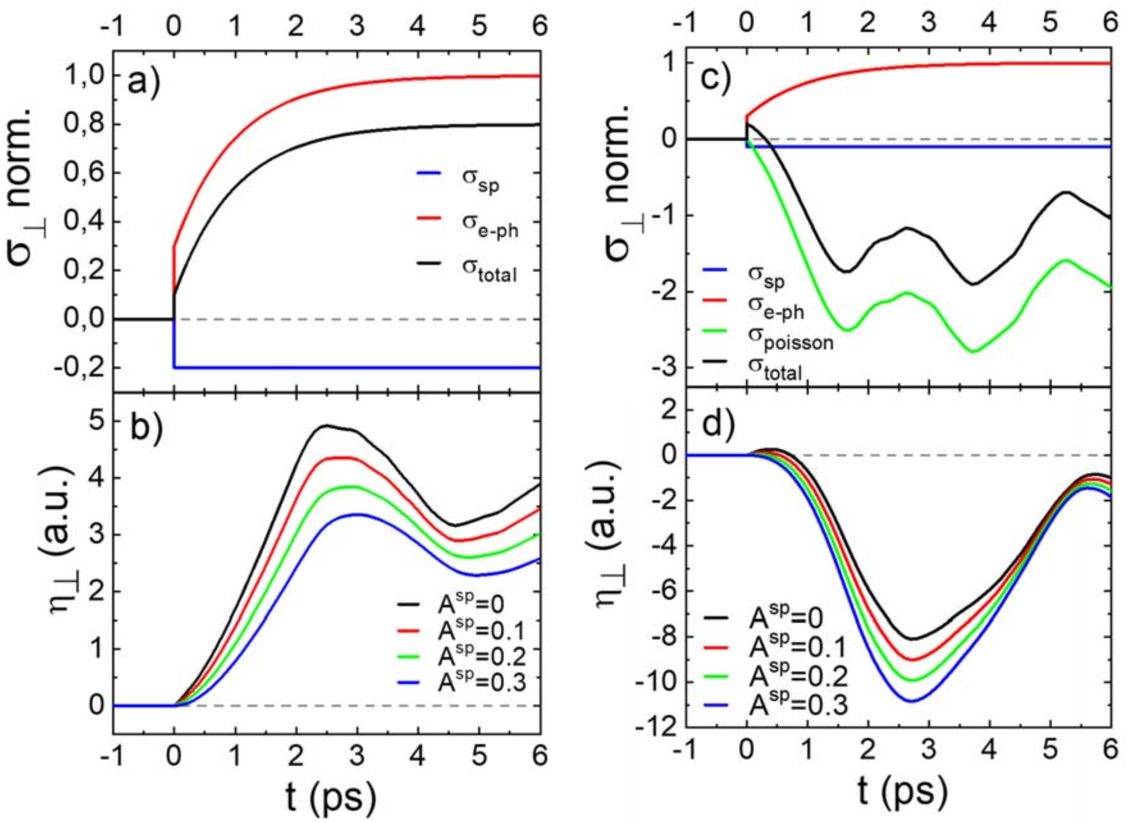

Figure S6: FEM modeling of the mechanical response of a continuous FePt film and a free FePt grain: a) time-dependent out of plane stresses $\sigma_{\perp}(t)$ acting on the FePt film for $A^{sp} = 0.2$ (solid lines). The dashed line shows the zero line corresponding to $\sigma_{sp} = 0$ for $A^{sp} = 0$ b) Average out of plane strain $\eta_{\perp}(t)$ in arbitrary units (a.u.) computed for various ratios A^{sp} of the spin and electron-phonon stress amplitudes. c-d) same a- b) for a free grain.

5) Laser based plasma x-ray source and femtosecond x-ray diffractometer

The principal components of the laser-based femtosecond plasma x-ray source (PXS) have remained unchanged since their installation nine years ago.[38] The input to the PXS are 50 fs laser pulses at a center wavelength of 800 nm with a pulse energy of 5 mJ at 1kHz

repetition rate that are focused by a 2 inch 90° off-axis parabolic mirror with a focal length of f = 10 cm into a vacuum chamber. At the focus, a 15 µm thick Cu band is transported fast enough to offer a fresh spot to each laser pulse impinging at a repetition rate of 1 kHz. The entrance and exit windows of the chamber are protected from the Cu plasma debris by moving plastic bands. The emitted x-rays are focused and monochromatized to the K_α doublet by a Montel multilayer optic with a convergence of 0.3° and a focal spot size of 200–300 µm FWHM at a distance d = 1000 mm from the PXS point source. The x-ray pulses contain only about 500-1000 photons per pulse on the sample. Key to the good signal to noise ratio are the gated CMOS 2D pixel detector and a careful optimization of the stability of the entire setup including the pump-laser. The x-rays are guided through evacuated tubes in order to avoid absorption and scattering by air molecules. The detector rests on the 2Θ arm of the 2-circle goniometer and detects scattered x-rays in the vicinity of the selected Bragg reflections, that are evaluated as reciprocal space mapping with a convergent beam[43].

Each data point in Figs. 1a,b), 2 and 3 took an acquisition time of about 120 s for the granular film and 70 s for the continuous film.

REFERENCES AND NOTES

1. E. F. Wasserman, Invar: Moment-volume instabilities in transition metals and alloys. *Handbook Ferromagn. Mater.* **5**, 237–322 (1990).

2. C. E. Guillaume, Recherches sur les aciers au nickel. Dilatations aux températures élevées; résistance électrique. *C. R. Acad. Sci.* ***125***, 18 (1897).

3. R. J. Weiss, The origin of the `Invar'effect. *Proc. Phys. Soc.* **82**, 281 (1963).

4. M. Van Schilfgaarde, I. A. Abrikosov, B. Johansson, Origin of the invar effect in iron-nickel alloys. *Nature* **400**, 46–49 (1999).

5. S. Khmelevskyi, I. Turek, P. Mohn, Large negative magnetic contribution to the thermal expansion in iron-platinum alloys: Quantitative theory of the invar effect. *Phys. Rev. Lett.* **91**, 037201 (2003).

6. C. Dornes, Y. Acremann, M. Savoini, M. Kubli, M. J. Neugebauer, E. Abreu, L. Huber, G. Lantz, C. A. F. Vaz, H. Lemke, E. M. Bothschafter, M. Porer, V. Esposito, L. Rettig, M. Buzzi, A. Alberca, Y. W. Windsor, P. Beaud, U. Staub, D. Zhu, S. Song, J. M. Glownia, S. L. Johnson, The ultrafast Einstein–de Haas effect. *Nature* **565**, 209–212 (2019).

7. E. Jal, V. López-Flores, N. Pontius, T. Ferté, N. Bergeard, C. Boeglin, B. Vodungbo, J. Lüning, N. Jaouen, Structural dynamics during laser-induced ultrafast demagnetization. *Phys. Rev. B.* **95**, 184422 (2017).

8. A. von Reppert, J. Pudell, A. Koc, M. Reinhardt, W. Leitenberger, K. Dumesnil, F. Zamponi, M. Bargheer, Persistent nonequilibrium dynamics of the thermal energies in the spin and phonon systems of an antiferromagnet. *Struct. Dyn.* **3**, 054302 (2016).

9. J. Pudell, A. von Reppert, D. Schick, F. Zamponi, M. Rössle, M. Herzog, H. Zabel, M. Bargheer, Ultrafast negative thermal expansion driven by spin disorder. *Phys. Rev. B.* **99**, 094304 (2019).

10. C. von Korff Schmising, A. Harpoeth, N. Zhavoronkov, Z. Ansari, C. Aku-Leh, M. Woerner, T. Elsaesser, M. Bargheer, M. Schmidbauer, I. Vrejoiu, D. Hesse, M. Alexe, Ultrafast magnetostriction and phonon-mediated stress in a photoexcited ferromagnet. *Phys. Rev. B.* **78**, 060404 (2008).

11. A. H. Reid, X. Shen, P. Maldonado, T. Chase, E. Jal, P. W. Granitzka, K. Carva, R. K. Li, J. Li, L. Wu, T. Vecchione, T. Liu, Z. Chen, D. J. Higley, N. Hartmann, R. Coffee, J. Wu, G. L. Dakovski, W. F. Schlotter, H. Ohldag, Y. K. Takahashi, V. Mehta, O. Hellwig, A. Fry, Y. Zhu, J. Cao, E. E. Fullerton, J. Stöhr, P. M. Oppeneer, X. J. Wang, H. A. Dürr, Beyond a phenomenological description of magnetostriction. *Nat. Commun.* **9**, 388 (2018).

12. A. Koc, M. Reinhardt, A. von Reppert, M. Rössle, W. Leitenberger, M. Gleich, M. Weinelt, F. Zamponi, M. Bargheer, Grueneisen-approach for the experimental determination of transient spin and phonon energies from ultrafast x-ray diffraction data: Gadolinium. *J. Phys. Condens. Matter* **29**, 264001 (2017).

13. E. Beaurepaire, J.-C. Merle, A. Daunois, J.-Y. Bigot, Ultrafast spin dynamics in ferromagnetic nickel. *Phys. Rev. Lett.* **76**, 4250–4253 (1996).

14. J. Kimling, J. Kimling, R. B. Wilson, B. Hebler, M. Albrecht, D. G. Cahill, Ultrafast demagnetization of FePt:Cu thin films and the role of magnetic heat capacity. *Phys. Rev. B.* **90**, 224408 (2014).

15. T. Kampfrath, A. Sell, G. Klatt, A. Pashkin, S. Mährlein, T. Dekorsy, M. Wolf, M. Fiebig, A. Leitenstorfer, R. Huber, Coherent terahertz control of antiferromagnetic spin waves. *Nat. Photonics* **5**, 31–34 (2011).

16. F. Hansteen, A. Kimel, A. Kirilyuk, T. Rasing, Nonthermal ultrafast optical control of the magnetization in garnet films. *Phys. Rev. B.* **73**, 014421 (2006).

17. J. W. Kim, M. Vomir, J. Y. Bigot, Controlling the spins angular momentum in ferromagnets with sequences of picosecond acoustic pulses. *Sci. Rep.* **5**, 8511 (2014).

18. M. Sander, J. E. Pudell, M. Herzog, M. Bargheer, R. Bauer, V. Besse, V. Temnov, P. Gaal, Quantitative disentanglement of coherent and incoherent laser-induced surface deformations by time-resolved x-ray reflectivity. *Appl. Phys. Lett.* **111**, 261903 (2017).

19. D. C. Heinecke, O. Kliebisch, J. Flock, A. Bruchhausen, K. Köhler, T. Dekorsy, Selective excitation of zone-folded phonon modes within one triplet in a semiconductor superlattice. *Phys. Rev. B* **87**, 075307 (2013).

20. T. Cheng, J. Wu, T. Liu, X. Zou, J. Cai, R. W. Chantrell, Y. Xu, Dual-pump manipulation of ultrafast demagnetization in TbFeCo. *Phys. Rev. B.* **93**, 064401 (2016).

21. K. Bühlmann, R. Gort, G. Salvatella, S. Däster, A. Fognini, T. Bähler, C. Dornes, C. A. F. Vaz, A. Vaterlaus, Y. Acremann, Ultrafast demagnetization in iron: Separating effects by their nonlinearity. *Struct. Dyn.* **5**, 044502 (2018).

22. J. Y. Shi, M. Tang, Z. Zhang, L. Ma, L. Sun, C. Zhou, X. F. Hu, Z. Zheng, L. Q. Shen, S. M. Zhou, Y. Z. Wu, L. Y. Chen, H. B. Zhao, Impact of ultrafast demagnetization process on magnetization reversal in $L1_0$ FePt revealed using double laser pulse excitation. *Appl. Phys. Lett.* **112**, 082403 (2018).

23. G. D. Barrera, J. A. O. Bruno, T. H. K. Barron, N. L. Allan, Negative thermal expansion. *J. Phys. Condens. Matter* **17**, R217–R252 (2005).

24. O. Mosendz, S. Pisana, J. W. Reiner, B. Stipe, D. Weller, Ultra-high coercivity small-grain FePt media for thermally assisted recording (invited). *J. Appl. Phys.* **111**, 07B729 (2012).

25. D. Weller, G. Parker, O. Mosendz, A. Lyberatos, D. Mitin, N. Y. Safonova, M. Albrecht, Review article: FePt heat assisted magnetic recording media. *J. Vac. Sci. Technol.* **34**, 060801 (2016).

26. K. Hono, Y. K. Takahashi, G. Ju, J.-U. Thiele, A. Ajan, X. Yang, R. Ruiz, L. Wan, Heat-assisted magnetic recording media materials. *MRS Bull.* **43**, 93–99 (2018).

27. A. von Reppert, L. Willig, J.-E. Pudell, M. Rössle, W. Leitenberger, M. Herzog, F. Ganss, O. Hellwig, M. Bargheer, Ultrafast laser generated strain in granular and continuous FePt thin films. *Appl. Phys. Lett.* **113**, 123101 (2018).

28. K. Sumiyama, M. Shiga, M. Morioka, Y. Nakamura, Characteristic magnetovolume effects in Invar type Fe-Pt alloys. *J. Phys. F Met. Phys.* **9**, 1665–1677 (1979).

29. Y. Tsunoda, H. Kobayashi, Temperature variation of the tetragonality in ordered PtFe alloy. *J. Magn. Magn. Mater.* **272–276**, 776–777 (2004).

30. R. Nicula, O. Crisan, A. D. Crisan, I. Mercioniu, M. Stir, F. Vasiliu, Thermal stability, thermal expansion and grain-growth in exchange-coupled Fe–Pt–Ag–B bulk nanocomposite magnets. *J. Alloys Compd.* **622**, 865–870 (2015).

31. P. Rasmussen, X. Rui, J. E. Shield, Texture formation in FePt thin films via thermal stress management. *Appl. Phys. Lett.* **86**, 191915 (2005).

32. P. W. Granitzka, E. Jal, L. Le Guyader, M. Savoini, D. J. Higley, T. Liu, Z. Chen, T. Chase, H. Ohldag, G. L. Dakovski, W. F. Schlotter, S. Carron, M. C. Hoffman, A. X. Gray, P. Shafer, E. Arenholz, O. Hellwig, V. Mehta, Y. K. Takahashi, J. Wang, E. E. Fullerton, J. Stöhr, A. H. Reid, H. A. Dürr, Magnetic switching in granular FePt layers promoted by near-field laser enhancement. *Nano Lett.* **17**, 2426–2432 (2017).

33. O. Hovorka, S. Devos, Q. Coopman, W. J. Fan, C. J. Aas, R. F. L. Evans, X. Chen, G. Ju, R. W. Chantrell, The Curie temperature distribution of FePt granular magnetic recording media. *Appl. Phys. Lett.* **101**, 052406 (2012).

34. L. Willig, A. Von Reppert, M. Deb, F. Ganss, O. Hellwig, M. Bargheer, Finite-size effects in ultrafast remagnetization dynamics of FePt. *Phys. Rev. B.* **100**, 224408 (2019).

35. J. M. D. Coey, *Magnetism and Magnetic Materials* (Cambridge Univ. Press, 2012).

36. A. Lyberatos, G. J. Parker, Model of ballistic-diffusive thermal transport in HAMR media. *Jpn. J. Appl. Phys.* **58**, 045002 (2019).

37. T. H. K. Barron, J. G. Collins, G. K. White, Thermal expansion of solids at low temperatures. *Adv. Phys.* **29**, 609–730 (1980).

38. D. Schick, A. Bojahr, M. Herzog, C. von Korff Schmising, R. Shayduk, W. Leitenberger, P. Gaal, M. Bargheer, Normalization schemes for ultrafast x-ray diffraction using a table-top laser-driven plasma source. *Rev. Sci. Instrum.* **83**, 025104 (2012).

39. M. Bargheer, N. Zhavoronkov, R. Bruch, H. Legall, H. Stiel, M. Woerner, T. Elsaesser, Comparison of focusing optics for femtosecond x-ray diffraction. *Appl. Phys. B.* **80**, 715–719 (2005).

40. T. Shima, K. Takanashi, Y. K. Takahashi, K. Hono, Preparation and magnetic properties of highly coercive FePt films. *Appl. Phys. Lett.* **81**, 1050–1052 (2002).

41. A. von Reppert, R. M. Sarhan, F. Stete, J. Pudell, N. Del Fatti, A. Crut, J. Koetz, F. Liebig, C. Prietzel, M. Bargheer, Watching the vibration and cooling of ultrathin gold nanotriangles by ultrafast x-ray diffraction. *J. Phys. Chem. C*, **120**, 28894–28899 (2016).

42. P. Tengdin, W. You, C. Chen, X. Shi, D. Zusin, Y. Zhang, C. Gentry, A. Blonsky, M. Keller, P. M. Oppeneer, H. Kapteyn, Z. Tao, M. Murnane, Critical behavior within 20 fs drives the out-of-equilibrium laser-induced magnetic phase transition in nickel. *Sci. Adv.* **4,** eaap9744 (2018).

43. D. Schick, R. Shayduk, A. Bojahr, M. Herzog, C. von Korff Schmising, P. Gaal, M. Bargheer, Ultrafast reciprocal-space mapping with a convergent beam. *J. Appl. Cryst.* **46**, 1372–1377 (2013).
